# Early Adolescents' Experiences During the COVID-19 Pandemic and Changes in Their Well-Being

**DOI:** 10.3389/fpubh.2022.823303

**Published:** 2022-05-03

**Authors:** Anne Gadermann, Kimberly Thomson, Randip Gill, Kimberly A. Schonert-Reichl, Monique Gagné Petteni, Martin Guhn, Michael T. Warren, Eva Oberle

**Affiliations:** ^1^Human Early Learning Partnership, School of Population and Public Health, University of British Columbia, Vancouver, BC, Canada; ^2^Department of Psychology, University of Illinois Chicago, Chicago, IL, United States

**Keywords:** well-being, mental health, adolescent, COVID-19, survey

## Abstract

**Background:**

Early adolescence is a time of psychological and social change that can coincide with declines in mental health and well-being. This study investigated the psychological and social impacts of the COVID-19 pandemic from the perspective of students who responded to a survey in Grades 7 and 8 (ages 12–14) in British Columbia (BC), Canada. The objectives of this study were (i) to provide an overview on early adolescents' experiences and social-emotional well-being during the pandemic; and (ii) to examine whether changes in social experiences as well as feeling safe from getting COVID-19 at school were associated with changes in well-being outcomes over the course of a year.

**Methods:**

A sample of *n* = 1,755 students from a large public school district self-reported on their life satisfaction, optimism, and symptoms of sadness across two time points: First, in their Grade 7 year (pre-pandemic; January to March, 2020) and then 1 year later in their Grade 8 year (during the pandemic; January to March, 2021). In Grade 8, students also reported on pandemic-specific experiences, including changes in mental health, social relationships, and activities, as well as coping strategies and positive changes since the pandemic. Data were collected online using the Middle Years Development Instrument (MDI), a population-based self-report tool that assesses children's social-emotional development and well-being in the context of their home, school, and neighborhood. Multivariable linear regression analyses were used to examine associations between pandemic-related changes in relationships and perceived safety from getting COVID-19 at school with changes in well-being outcomes.

**Results:**

Students reported a range of experiences, with a large proportion reporting moderate concerns and impacts of the pandemic, including worries about their mental health and missing school activities. Students reported significantly lower optimism, lower life satisfaction, and higher sadness compared to the previous year. Within the sample, improvements in relationships with parents and other adults at home was associated with higher well-being during the pandemic.

**Implications:**

Results from this study can inform decision making of policy-makers, educators, and practitioners working with youth, by providing information on students' experiences during the pandemic and identifying factors that may be protective for students' mental health during and beyond the pandemic.

## Introduction

The COVID-19 pandemic has led to profound changes to the social worlds of students, including disruptions within family life, school closures, physical distancing measures within schools upon re-opening, and cancelation of most in-person social activities. These disruptions have led many researchers, physicians, and educators to raise concerns regarding the impact of the pandemic and related restrictions on students' mental health and well-being, particularly because of adolescents' sensitivity to social contexts during this developmental window (1–5). In addition to the potential of immediate mental health impacts, the pandemic may have long-term impacts on adolescents' physical, social, and emotional health due to increased stress during a period when the physiological systems that regulate and respond to stress are still developing (6). For example, social isolation is a stressor shown to be adversely associated with children's developing stress response systems (7).

The early adolescent years are a time of psychological and social change associated with the onset of many mental health problems and declines in well-being (8–10). During this time, individuals are developing a greater sense of self-awareness, awareness of others, and identity formation, as well as experiencing the onset of puberty, encountering social changes within friendship groups, and experiencing greater academic expectations and challenges (11–14). Pre-pandemic research documents the important stress-buffering effects of social relationships (15). However, in the context of the COVID-19 pandemic, it is unclear how social isolation and changes within homes and schools have affected social relationships, and how these changes, in turn, have affected students' mental health. The purpose of the current study was to investigate 12 to 14 year-old students' experiences during the pandemic after ~4 months back at school. A core interest was examining what factors were negatively associated with students' mental health and—from a strengths-based lens—what factors were associated with resilience. This research was informed by the Positive Youth Development (PYD) framework [see (16)]. Consistent with PYD, this study takes a strengths-based perspective on child and youth development by examining assets and protective factors in several developmental contexts (e.g., school, home, community) that contribute to positive outcomes in development.

### Pandemic-Related Changes in Adolescent Mental Health

Research studies worldwide have documented declines in children's and adolescents' mental health associated with the COVID-19 pandemic (1–5, 17, 18). Among adolescents ages 13 to 17, a review of COVID-19 studies including validated and designed-for-purpose self-report measures documented increases in depression, anxiety, and stress (4). In Germany, 40% of 11- to 17 year-olds reported worsened mental health due to the pandemic, 18% reported more mental health problems, and 24% reported higher anxiety (5). In Israel, a repeated-measures study conducted with 11 to 17 year-olds before and during the pandemic found adolescents were reporting increased depression, anxiety, and panic, and decreased positive emotions and life satisfaction (19). Similarly in Australia, a repeated measures study conducted with 13 to 16 year-olds found that adolescents reported increased depression and anxiety and decreased life satisfaction during the first 2 months of the pandemic compared to the previous year (20). This study found that increased conflict with parents was associated with increases in mental health problems (20). Research in Canada with early adolescents aged 10 to 12 years old using self-report items adapted from the National Institutes of Mental Health CoRonavIruS Health Impact Survey (CRISIS) found that increased stress associated with social isolation was associated with mental health problems (1). Specifically, adolescents reported increased depression (35%), anxiety (40%), irritability (45%), attention problems (46%), and hyperactivity (42%) (1).

However, these studies have also shown that the COVID-19 pandemic has not affected all adolescents in the same way. In the Canadian study using the CRISIS measure, 9% to 13% of participants reported improvements in mental health. In a national poll of 10 to 17 year-olds conducted during school closures 71% of Canadian students reported feeling bored, 54% reported missing their friends, and 41% reported feeling “quite normal” (21). In a repeated-measures study of students in grades 6 to 12 in the United States, students reported on average that their mental health improved during the first 3 months of the pandemic compared to the previous year; possible explanations for this improvement include reduced academic pressure (22). However, the authors also noted that improvements were not reported by all students. The wide ranges in mental health outcomes prompt questions regarding what changes in social experiences predict adolescents' mental health during the pandemic, and what contextual factors promote resilience during times of crisis.

### Pandemic-Related Changes in Early Adolescents' Social Contexts

According to the PYD framework, thriving in adolescence is supported by the presence of developmental assets that are internal (e.g., positive values) and external (e.g., social support and activities that provide opportunities for engagement, leadership, and success) (16). Importantly, it is the interaction between individuals and their contexts that drives positive development (16). The COVID-19 pandemic has changed these developmental opportunities in several ways. For example, for some adolescents, the pandemic caused significant changes within the home environment. Families have endured enormous pressures due to the pandemic, including unemployment, financial pressure, relationship challenges, and increased caregiving and homeschooling responsibilities (2, 23, 24). Since the start of the pandemic, parents have reported more frequent negative mood and worsened mental health (2), as well as increases in negative parent-child interactions including increased conflicts and discipline (23–26). At the same time, some studies found that parents reported increases in positive interactions with children during the pandemic including increased closeness and showing love and affection, perhaps because of more time for conversations and shared activities (23, 25–27). There are also wide socio-economic disparities among families, which impact the opportunities parents have to provide activities for their children at home, including access to online school-based activities and social networks (28). In summary, the available evidence points to a general trend toward greater stress and worse home environments during the pandemic, especially for families experiencing marginalization or disadvantages, with the caveat that this unique period may also have afforded opportunities for closer family relationships.

Another significant change occurred within the school environment. In the past year, students worldwide became more isolated due to school closures, canceled activities, and limits to social group sizes upon the return to school. Specifically in British Columbia, Canada, schools were closed mid-March 2020 with learning activities moved online (29). Schools partially re-opened in June 2020 before closing again at the end of June for summer break, and all students returned to a modified school setting in September 2020 (29). Starting from September 2020, BC schools operated under the guidelines of the Provincial Health Officer that mandated several infection prevention and exposure control measures including moving desks and implementing physical barriers to avoid close contact, staggering lunch and recess, assigning students to a specific cohort up to 60 people with whom all activities were conducted, maintaining physical distancing and hand hygiene, and limiting school gatherings such as school assemblies and extracurricular activities (30). To date, there is limited research on early adolescents' mental health in the context of schools re-opening. On the one hand, students who returned to school continued to face uncertainty, altered social routines, and potential health concerns related to virus exposure (31). On the other hand, the return to school brought greater opportunities to see friends and teachers in person and the potential to access school mental health supports. Existing research on the return to school found that most students ages 12 to 18 years old reported low levels of COVID-19-related stress (e.g., constantly thinking about COVID-19, sleep problems), however stress was higher among girls and older students (31). Other reports indicated that a higher proportion of students attending school during the pandemic reported lower well-being compared to previous academic years (32).

### Protective Factors for Adolescent Mental Health

Studies that have investigated protective factors of adolescents' mental health and well-being before and during the COVID-19 pandemic provide important information. The PYD framework suggests that positive social relationships are a key foundation for promoting adolescent mental health (16). Supportive social contexts and high-quality relationships have also been identified as key components of resilience (i.e., competence in the face of adversity) and promoters of competence (i.e., when no adversity is present) (33). During the pandemic, adolescents ages 13 to 18 years-old who reported spending more time with family also reported less loneliness and depression (34). Similarly, among younger adolescents ages 9 to 11 years-old, connectedness to adults at home was associated with lower depression and anxiety during the pandemic as well as greater happiness (35). Interestingly, in this same study, connectedness to peers was not associated with mental health and well-being outcomes, which was explained as potentially resulting from the limited opportunities students had to interact with peers during the study period. In other research, students have furthermore identified potential positive impacts of the pandemic, including more time for activities they previously were too busy to pursue, increased exercise, and increased control over one's life (36). In two repeated-measures studies, social connectedness, perceived social support and consistent daily routines were also identified as protective factors for adolescent mental health during the pandemic (19, 20). Remaining gaps in research include a limited understanding of the proportion of adolescents who have maintained positive relationships during the pandemic and to what extent pandemic-related changes in relationships with adults and peers are associated with mental health and well-being in the context of returning to school.

### The Current Study

The current study capitalized on a unique dataset that linked survey responses from a population cohort of students attending public school in one of the largest school districts in British Columbia, Canada, from Grade 7 (January-March 2020; just prior to province-wide restrictions due to the COVID-19 pandemic) to Grade 8 (January-March 2021; nearly 1 year into the pandemic and ~4 months after schools re-opened). In Grade 8, students answered questions on their experiences during the pandemic, including changes in activities and social relationships, as well as their mental health and perceived safety from getting the virus at school. In this study, we addressed two research objectives: (i) to provide an overview on early adolescents' experiences and social-emotional well-being during the pandemic; (ii) to examine whether changes in social experiences as well as feeling safe from getting COVID-19 at school were associated with changes in well-being outcomes over the course of a year.

## Methods

### Participants

All enrolled Grade 7 and 8 public middle school students from a large urban school district in British Columbia (BC), Canada, were invited to participate in the study at two time points. Compared to the average socio-economic characteristics of households in BC, this district has a comparable but slightly higher median household income and higher proportion of households considered low-income (37). Time 1 data were collected in Grade 7 (pre-pandemic; January-March 2020)^1^ and Time 2 data were collected 1 year later in Grade 8 (~10–12 months after the COVID-19 pandemic was first declared; January-March 2021). At both time points, all 14 middle schools of this district took part in the study. All participating students in this study attended school in person at Time 1 and Time 2. In Grade 7, 2,214 students participated in the survey, representing 86% of the district's Grade 7 public school population. In Grade 8, 2,131 students participated in the survey, representing 81% of the district's Grade 8 public school population. A total of 1,755 students had linkable data across the two time points and were included in this study (49.2% girls, 50.8% boys). In the linked sample, mean age at Time 1 (Grade 7) was 13.0 years, SD = 0.12. Mean age at Time 2 (Grade 8) was 14.0 years, SD = 0.12. Overall, 56.6% of the students reported “English only” as the first language learned at home, 18.9% reported “English and another language”, and 24.5% reported a language other than English as the first language learned at home. The most common first languages learned other than English were Korean (23%), Mandarin (22%), and Cantonese (9%). A comparison between children in the linked sample and those with data limited to Grade 7 found no differences with regard to age, gender, or English as a second language, but that children in the linked sample generally reported higher connectedness to adults and peer belonging than children lost to follow up.

### Procedure

At both Time 1 and Time 2, data were collected using the Middle Years Development Instrument (MDI), a validated self-report measure of social and emotional competencies for children and adolescents measuring their well-being, health, and developmental assets (39, 40). All students within Grades 7 (Time 1) and 8 (Time 2) in participating schools were invited to participate, with the study team providing parents/guardians 4 weeks' notice to inquire about the study and opt their children out of participation. Schools and classroom teachers could additionally opt-out of participation. Prior to the survey, students were read an assent script and were provided the choice to do an alternative activity to the survey that would not identify their non-participation. At both time points, survey data were collected via an online survey, conducted at school during school hours^2^. At Time 2 (Grade 8), students completed an additional survey module that asked about pandemic-specific experiences, including changes in mental health, social relationships, and activities, as well as coping strategies and positive changes since the pandemic. Ethics approval for both surveys and data linkage was obtained from the University of British Columbia Research Ethics Board. Data linkage was completed at Population Data BC using children's Personal Education Number and child date of birth.

### Measures

#### Well-Being Outcomes

Data were collected using the MDI self-report survey for children aged 9–14 (40). The MDI includes 101 items and subscales that have been previously used in developmental research with children and/or adolescents. Items were initially pilot tested in focus groups with children to ensure clarity and understanding at a Grade 4 level (40). Previous research has found the MDI to have satisfactory psychometric properties, with good internal consistency and convergent validity evidence for subscales (39, 40). The MDI has been implemented across Canada and has been validated with child and early adolescent populations internationally (42, 43). A copy of the MDI survey as well as district reports for previous Grade 7 years are available from http://earlylearning.ubc.ca/mdi/.

Children's self-reported well-being (life satisfaction, optimism) and internalizing (sadness) were measured at Time 1 and Time 2 using three subscales of the MDI (40). On each subscale, students rated their agreement with a series of statements using a 5-point Likert type response format (1 = Disagree a lot, 5 = Agree a lot). *Optimism* (3 items) was adapted from the previously validated *Optimism Resiliency Inventory Subscale* (44). In Grade 8, the mean scale score was 3.47 (standard deviation (SD) = 0.96). *Satisfaction with Life* (5 items) was measured using the *Satisfaction with Life Scale adapted for Children* (SWLS-C) (45, 46), a validated children's measure based on the *Satisfaction with Life Scale* for adults (47). In Grade 8, the mean scale score was 3.55 (SD = 1.00). *Sadness* (3 items) was adapted from the previously validated *Seattle Personality Questionnaire for Young School-Age Children* (48). In Grade 8, the mean scale score was 3.00 (SD = 1.01). Each subscale had good internal consistency, with Cronbach's alpha in Grade 8 of 0.81, 0.89, and 0.78 for optimism, life satisfaction, and sadness, respectively. These were comparable with Cronbach's alphas for optimism, life satisfaction, and sadness in Grade 7 of 0.81, 0.87, and 0.77.

#### Pandemic-Related Experiences

In 2021, the study team developed a subset of 18 items specifically to measure the impact of the COVID-19 pandemic and related restrictions on early adolescents. Similar to the development of the MDI, pandemic-related items were selected and adapted from existing, previously validated surveys where possible, based on their contextual and developmental relevance. Other items were developed for the purpose of this study by researchers with input from educators and students. Survey items were piloted with children and stakeholders from schools and the BC Ministry of Education and refined based on their feedback. These items are described below and provided in Appendix A. The following five constructs measure pandemic-specific experiences and were therefore measured only at Time 2 (January to March, 2021).

##### Worries About Mental Health

Students' worries about mental health were assessed using an item adapted from the National Institutes of Mental Health CRISIS questionnaire V0.3 youth self-report baseline form, “During the PAST TWO WEEKS, how worried have you been about your Mental/Emotional health being influenced by the COVID-19 pandemic?” This item was rated on a 5-point response format (1 = Not at all, 5 = Extremely) (49).

##### School-Based Public Health Practices and Perceived Safety From Getting COVID-19 at School

Students were given a list of common school-based public health practices, including “washing hands more frequently” and “practicing physical distancing.” Students rated how easy or hard these practices were on a 5-point response format (1 = Very hard, 5 = Very easy). These items were adapted from the general population British Columbia COVID-19 SPEAK survey developed by the British Columbia Centre for Disease Control (41). Additionally, students were asked, “How often do the COVID-19 safety measures at your school make you feel safe from getting COVID-19?” Items were rated on a 4-point response format (1 = Never safe, 4 = Always safe). This item was created specifically for this study with input from stakeholders.

##### Changes in Relationships Due to the Pandemic

Students were asked, “From before the COVID-19 pandemic to now, have your relationships with […] gotten better or worse?” This question was repeated for three relationship types: parents or other adults at home, teachers or other adults at school, and friends. Items were rated on a 5-point Likert type response format (1 = A lot worse, 5 = A lot better). These items were adapted from the CRISIS questionnaire V0.3 youth self-report baseline form, which originally asked participants to report how the quality of their relationships had changed due to the Coronavirus/COVID-19 crisis in the past 2 weeks, using the same rating scale.

##### Changes in Time Use

Students were asked, “How did the following change from before the COVID-19 pandemic to now?” Four items measured spending time with friends online, time with friends in person/face-to-face, time with family members who live in my home, and time outdoors (for example, playing sports, hiking, biking, going for walks). Students rated changes in time use on a 5-point Likert type response format (1 = Much less, 5 = Much more). These items were created specifically for this study by the research team with input from stakeholders.

##### Coping With Stress, Activities Missed at School, and Positive Changes Since the Pandemic

Students were asked, “What has helped you deal/cope with worries and stress related to the COVID-19 pandemic?” Students could select multiple options that applied, from a provided list. This item was adapted from a COVID-19 self-report mental health survey originally developed by the Mental Health Foundation in the United Kingdom (50). Students were also asked, “What are you missing the most at school during the COVID-19 pandemic?” and “What are some positive changes that have occurred in your life during the COVID-19 pandemic?” These items and answer options were created specifically for this study by the research team with input from stakeholders.

#### Gender

Student's gender was measured through school district recorded information (girls were coded as 1, boys as 2).

### Analyses

Frequencies were calculated for all measures examining self-reported changes in relationships, time use, and perceived safety from getting COVID-19 at school. Paired *t*-tests were conducted to measure unadjusted changes in optimism, life satisfaction, and sadness from Grade 7 (prior to the pandemic) to Grade 8. We conducted three multivariable linear regression models, entering groups of variables in blocks, to examine associations between pandemic-related changes in relationships and perceived safety from getting COVID-19 at school with well-being outcomes (optimism, life satisfaction, and sadness), adjusted for gender, age, born in Canada (yes/no), and previously reported well-being in Grade 7. We also calculated the PRATT-index for each predictor variable in the regression model to assess each variable's relative importance (51). The PRATT-index calculates the percentage of the total variance explained in the model that is explained by each predictor variable. The formula includes each predictor's beta weight, its correlation with the outcome variable, and the total *R*^2^ in the model [*d* = (ß * *r*xy)/*R*^2^] (51). Each variable receives a score from 0 to 1, with all variables taken together accounting for 100% of the variance explained in the model. Using criteria previously established by Thomas (52), predictors are considered relatively unimportant if *d* < 1/(2^*^p) where *p* is the total number of predictors in the model. In the full regression models within the current study, predictors with a PRATT-index score smaller than 0.06 [*d* < 1/(2^*^8)] explained relatively little of the variance in the model (<6% of the *R*^2^). This was a complete case analysis. Missing data were excluded listwise in the multiple regression analyses resulting in denominators of *n* = 1,569 for optimism, *n* = 1,549 for sadness, and *n* = 1,519 for life satisfaction.

## Results

### Mental Health and Perceived Safety of Getting COVID-19 at School During the Pandemic

**Figure 1:**
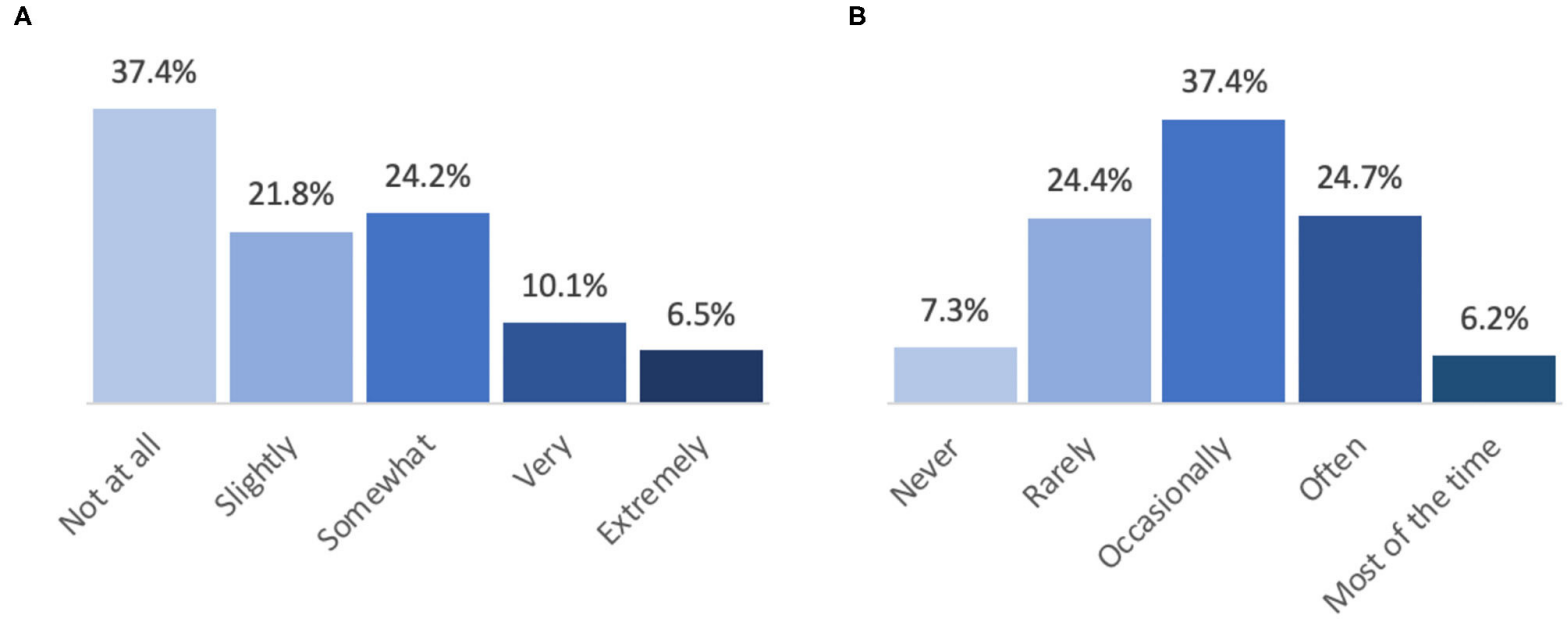
Students' self-reported mental health and frequency of reading or talking about the COVID-19 pandemic during the past 2 weeks. **(A)** During the past 2 weeks, how worried have you been about your mental/emotional health being influenced by the COVID-19 pandemic? **(B)** How much are you reading or talking about the COVID-19 pandemic?

Figure 1A presents the proportion of students who reported being worried about their mental/emotional health being influenced by the pandemic in the past 2 weeks. Nearly half of students (46%) reported feeling slightly or somewhat worried, and an additional 17% of students reported feeling very or extremely worried. When asked how much students were reading or talking about the COVID-19 pandemic (Figure 1B), the most frequent response (37%) was “occasionally.”

**Figure 2:**
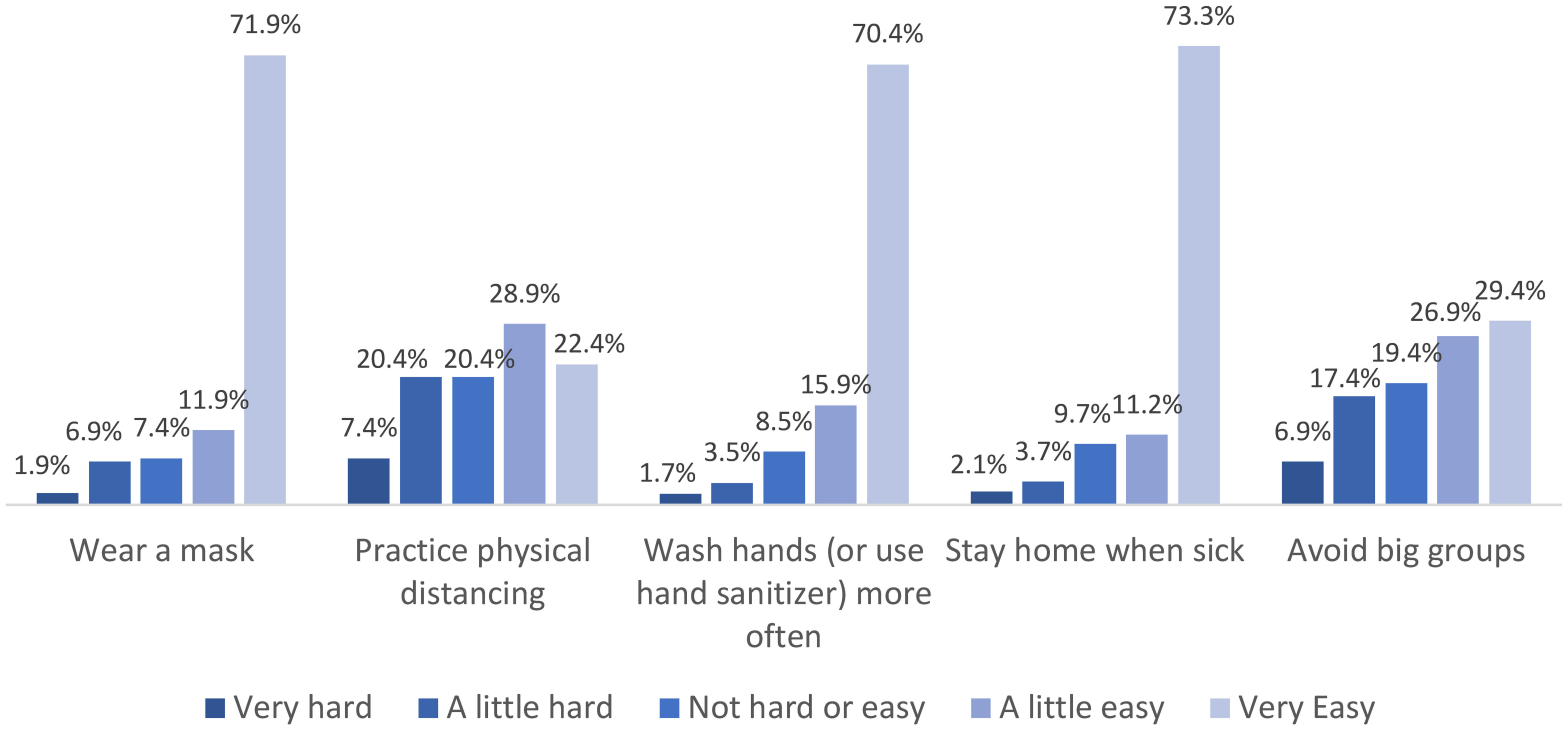
Students' self-reported ease of practicing COVID-19 safety measures at school.

At the time of data collection, school districts had put in place several safety measures to prevent the spread of COVID-19, including requiring face masks to be worn indoors, practicing physical distancing, washing hands and using hand sanitizer, canceling activities involving large groups, and requiring that students stay home when sick. Overall, the majority of students reported that it was a little easy or very easy to follow these safety protocols (Figure 2). The most challenging protocols to follow were practicing physical distancing and avoiding big groups. With these safety measures in place, 14% of students always felt safe from getting COVID-19 at school, 47% reported feeling safe most of the time, 31% reported feeling safe some of the time, and 8% never felt safe.

### Pandemic-Related Changes in Relationships

**Figure 3:**
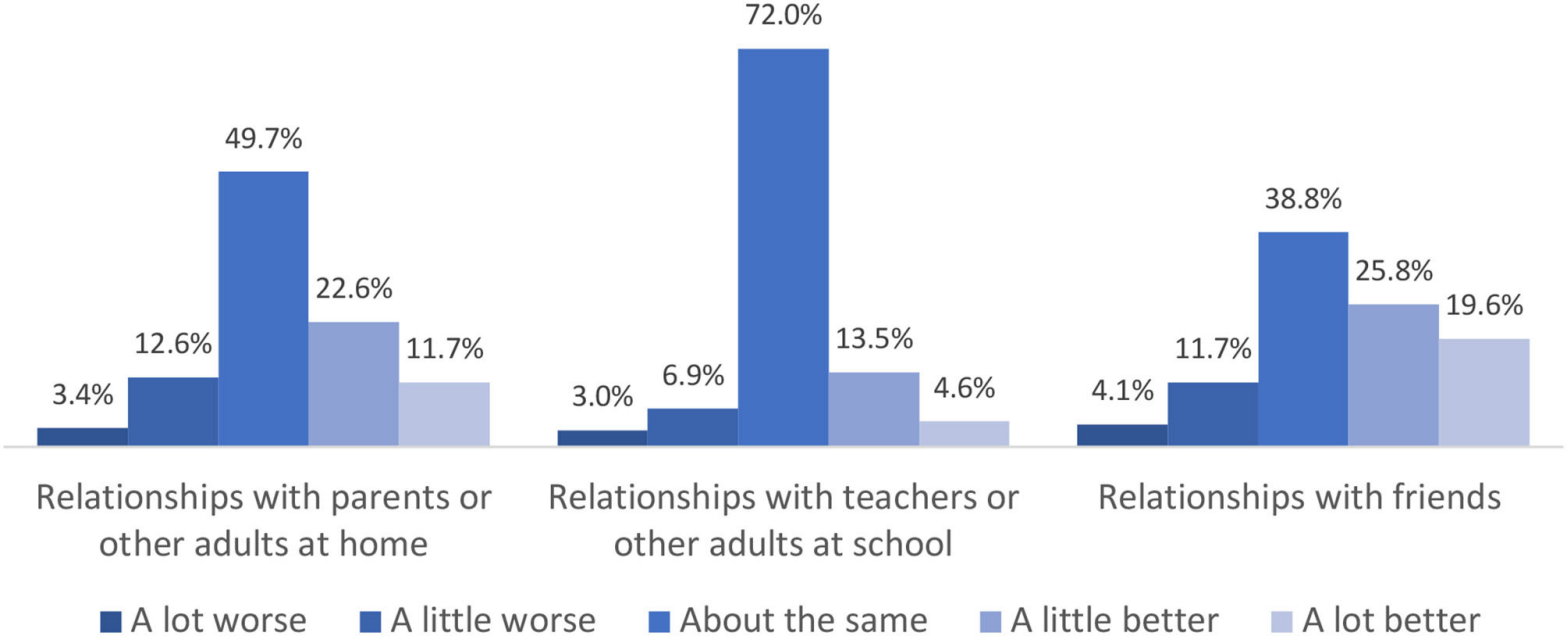
Students' perceived changes in relationships from before the COVID-19 pandemic to now.

Compared to prior to the COVID-19 pandemic, the majority of students reported that their relationships with parents (or other adults at home) and teachers (or other adults at school) had stayed the same or improved (Figure 3). Perceived relationship improvements were even more pronounced for friendships, in which 45% of students reported that relationships with friends had gotten a little or a lot better. In contrast, 16% of students reported that their relationships with parents or other adults at home and friends had gotten a little or a lot worse, and 10% reported worsened relationships with teachers or other adults at school.

### Pandemic-Related Changes in Activities and Time Use

Students reported several pandemic-related changes in their activities and time use. Compared to before the pandemic, 69% of students reported spending more or much more time with their friends online. Seventy-two percent reported spending less or much less time with friends in person/face-to-face. In contrast, 59% of students reported spending more or much more time with family members who live in their home. Thirty-two percent of students reported spending more or much more time outdoors, whereas 35% of students reported spending less or much less time outdoors.

**Figure 4:**
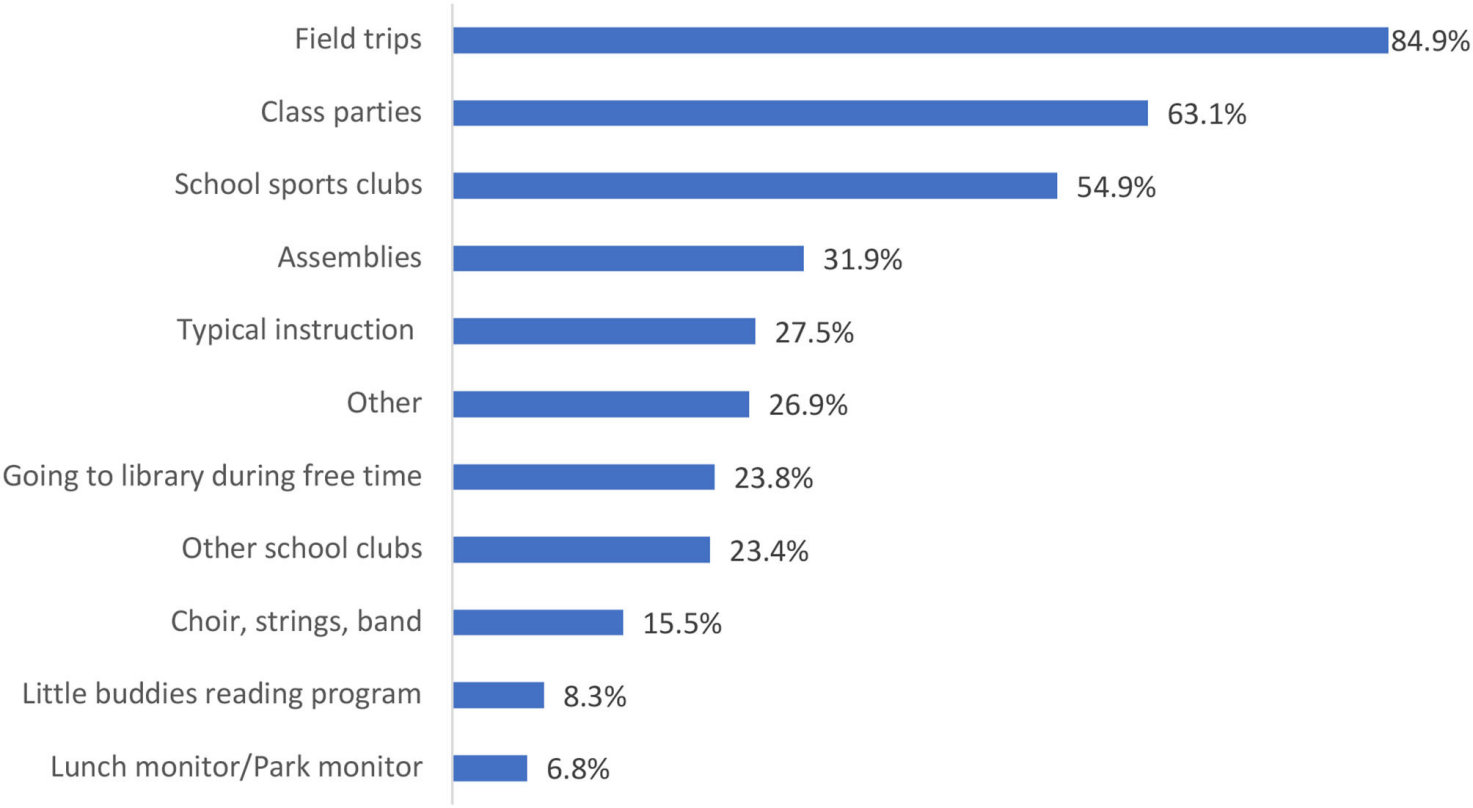
Activities that students missed most at school during the COVID-19 pandemic.

At the time of data collection, schools were in session but several restrictions were in place including canceled group activities, and learning within small working groups (29). When asked what students missed most at school during the COVID-19 pandemic, the most frequent response was field trips (85%), followed by class parties (63%), school sports clubs (55%), assemblies (32%), and typical instruction (28%) (Figure 4). Students also identified positive changes that had occurred in their lives during the pandemic, including having more time to themselves (58%), exploring other interests (44%), spending more time with family members (42%), getting more sleep (32%), spending more time with friends (30%), and helping others more (9%). However, 1 in 5 students (21%) reported that there were no positive changes.

**Figure 5:**
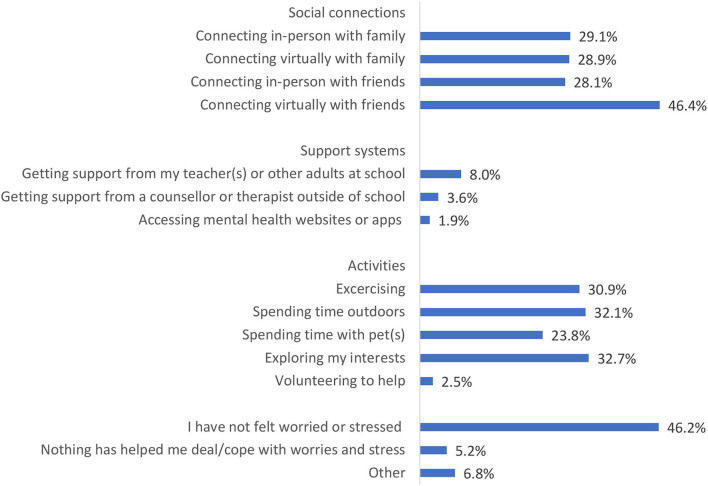
Strategies that helped students cope with worries and stress related to the pandemic in the past 2 weeks.

Students were also asked what had helped them deal/cope with worries and stress related to the COVID-19 pandemic. As shown in Figure 5, the most frequent response was connecting virtually with friends (46%), followed by exploring their interests (33%), spending time outdoors (32%), and exercising (31%). Forty-six percent of students reported that they had not felt worried or stressed due to the pandemic, and 5% reported that nothing had helped them deal/cope with worries or stress.

### Associations Between Pandemic-Related Changes and Well-Being

#### Overall Changes in Well-Being Compared to Before the Pandemic

Paired-samples *t*-tests identified that across all measures, self-reported well-being declined from prior to the pandemic (Grade 7) to during the pandemic (Grade 8). On average, students reported a 0.15 point decrease in mean optimism scores, *t*_(1,686)_ = −6.71, *SD*_mean_ = 0.91, Cohen's *d* = 0.16 [95% CI: 0.12,0.21], *p* < 0.001. Students reported a 0.21 point decrease in mean life satisfaction scores, *t*_(1,629)_ = −10.11, *SD*_mean_ = 0.86, Cohen's *d* = 0.25 [95% CI: 0.20,0.30], *p* < 0.001. Students' also reported 0.16 point increase in mean sadness scores, *t*_(1,669)_ = 6.92, *SD*_mean_ = 0.95, Cohen's *d* = 0.17 [95% CI: 0.12,0.22], *p* < 0.001.

#### Associations Between Pandemic-Related Changes and Optimism

Multivariable regression models were conducted to examine the associations between pandemic-related changes in relationships and context and early adolescents' well-being across three outcomes: optimism, life satisfaction, and sadness^3^. Table 1 presents results from multivariable regression models examining associations between child demographics, students' reported optimism in Grade 7, and pandemic-related changes in students' relationships and feelings of safety from getting COVID-19 at school with students' self-reported optimism in Grade 8. In the fully adjusted model with all variables entered simultaneously (model 3), higher optimism in Grade 8 was predicted by gender (boys higher optimism than girls; *B* = 0.14, 95% CI = 0.06, 0.21), higher optimism in Grade 7 (*B* = 0.48, 95% CI = 0.44, 0.52), improved relationships with adults at home (*B* = 0.17, 95% CI = 0.13, 0.22), improved relationships with adults at school (*B* = 0.09, 95% CI = 0.04, 0.15), improved relationships with friends (*B* = 0.05, 95% CI = 0.01, 0.09), and feeling safe from getting COVID-19 at school (*B* = 0.14, 95% CI = 0.09, 0.19). The Pratt Index identified three of these variables to be the most important in predicting students' Grade 8 optimism: optimism in Grade 7 (explaining 67% of the 37% total variance explained by the model), improvements in relationships with adults at home (16%), and feeling safe from getting COVID-19 at school (7%).

**Table 1:** Multivariable linear regression analysis predicting optimism score at Grade 8 (*n* = 1,569).

		**B**	**SE**	**β**	**95% CI**	**PRATT**
Model 1 Adjusted *R*^2^ = 0.01	Gender	0.21^***^	0.05	0.11	0.11, 0.30	
	Age	0.29	0.22	0.03	−0.14, 0.72	
	Born in Canada	−0.01	0.06	0.00	−0.12, 0.11	
Model 2 Adjusted *R*^2^ = 0.30	Gender	0.20^***^	0.04	0.10	0.12, 0.27	
	Age	0.17	0.18	0.02	−0.18, 0.53	
	Born in Canada	−0.05	0.05	−0.02	−0.14, 0.05	
	Grade 7 optimism	0.56^***^	0.02	0.54	0.51, 0.60	
Model 3 Adjusted *R*^2^ = 0.37	Gender	0.14^***^	0.04	0.07	0.06, 0.21	0.02
	Age	0.18	0.17	0.02	−0.16, 0.53	0.00
	Born in Canada	−0.03	0.05	−0.01	−0.12, 0.06	0.00
	Grade 7 optimism	0.48^***^	0.02	0.46	0.44, 0.52	0.67
	Change in home adult relationships	0.17^***^	0.02	0.17	0.13, 0.22	0.16
	Change in school adult relationships	0.09^**^	0.03	0.07	0.04, 0.15	0.04
	Change in friendships	0.05^*^	0.02	0.06	0.01, 0.09	0.03
	Feeling safe from getting COVID-19 at school	0.14^***^	0.02	0.12	0.09, 0.19	0.07

* *p < 0.05*

** *p < 0.01*

*** *p < 0.001*.

*B, unstandardized regression coefficient; SE, standard error of B; CI, confidence interval for B; β, beta weight; standardized regression coefficient. Gender (girl = 1, boy = 2) and Born in Canada (no = 0, yes = 1)*.

#### Associations Between Pandemic-Related Changes and Life Satisfaction

Table 2 presents results from multivariable regression models examining associations between predictor variables and students' self-reported life satisfaction in Grade 8. In the fully adjusted model, higher life satisfaction in Grade 8 was predicted by gender (boys higher life satisfaction than girls; *B* = 0.15, 95% CI = 0.07,0.22), higher life satisfaction in Grade 7 (*B* = 0.58, 95% CI = 0.53,0.62), improved relationships with adults at home (*B* = 0.20, 95% CI = 0.16,0.25), improved relationships with adults at school (*B* = 0.10, 95% CI = 0.04, 0.16), and feeling safe from getting COVID-19 at school (*B* = 0.11, 95% CI = 0.06,0.15). The Pratt Index identified two variables to be the most important in predicting students' Grade 8 life satisfaction: their life satisfaction in Grade 7 (explaining 72% of the 44% total variance explained by the model), and improvements in relationships with adults at home (16%).

**Table 2:** Multivariable linear regression analysis predicting satisfaction with life score at Grade 8 (*n* = 1,519).

		**B**	**SE**	**β**	**95% CI**	**PRATT**
Model 1 Adjusted *R*^2^ = 0.02	Gender	0.28^***^	0.05	0.14	0.18, 0.38	
	Age	0.07	0.23	0.01	−0.37, 0.51	
	Born in Canada	0.07	0.06	0.03	−0.05, 0.20	
Model 2 Adjusted *R*^2^ = 0.37	Gender	0.20^***^	0.04	0.10	0.12, 0.28	
	Age	0.10	0.18	0.01	−0.25, 0.45	
	Born in Canada	−0.11^*^	0.05	−0.05	−0.21, −0.01	
	Grade 7 satisfaction with life	0.65^***^	0.02	0.60	0.61, 0.69	
Model 3 Adjusted *R*^2^ = 0.44	Gender	0.15^***^	0.04	0.08	0.07, 0.22	0.02
	Age	0.10	0.17	0.01	−0.24, 0.44	0.00
	Born in Canada	−0.08	0.05	−0.03	−0.17, 0.02	0.00
	Grade 7 satisfaction with life	0.58^***^	0.02	0.53	0.53, 0.62	0.72
	Change in home adult relationships	0.20^***^	0.02	0.19	0.16, 0.25	0.16
	Change in school adult relationships	0.10^***^	0.03	0.07	0.04, 0.16	0.04
	Change in friendships	0.03	0.02	0.03	−0.01, 0.07	0.01
	Feeling safe from getting COVID-19 at school	0.11^***^	0.02	0.09	0.06, 0.15	0.04

* *p < 0.05*

**p < 0.01

*** *p < 0.001*.

*B, unstandardized regression coefficient; SE, standard error of B; CI, confidence interval for B; β, beta weight; standardized regression coefficient. Gender (girl = 1, boy = 2) and Born in Canada (no = 0, yes = 1)*.

#### Associations Between Pandemic-Related Changes and Sadness

Table 3 presents results from multivariable regression models examining associations between predictor variables and students' self-reported sadness in Grade 8. In the fully adjusted model, lower sadness in Grade 8 was predicted by gender (boys lower sadness than girls; *B* = −0.25, 95% CI = −0.33, −0.17), lower sadness in Grade 7 (*B* = 0.51, 95% CI = 0.47,0.55), improved relationships with adults at home (*B* = −0.17, 95% CI = −0.22, −0.12), and feeling safe from getting COVID-19 at school (*B* = −0.13, 95% CI = −0.18, −0.08). The Pratt Index identified three variables to be the most important in predicting students' Grade 8 sadness: gender (explaining 7% of the 37% total variance explained by the model), sadness in Grade 7 (75%), and improvements in relationships with adults at home (12%).

**Table 3:** Multivariable linear regression analysis predicting sadness score at Grade 8 (*n* = 1,549).

		**B**	**SE**	**β**	**95% CI**	**PRATT**
Model 1 Adjusted *R*^2^ = 0.04	Gender	−0.40^***^	0.05	−0.20	−0.50, −0.30	
	Age	0.02	0.23	0.00	−0.43, 0.48	
	Born in Canada	−0.03	0.06	−0.01	−0.15, 0.09	
Model 2 Adjusted *R*^2^ = 0.33	Gender	−0.29^***^	0.04	−0.15	−0.37, −0.21	
	Age	−0.01	0.20	0.00	−0.39, 0.38	
	Born in Canada	−0.04	0.05	−0.01	−0.14, 0.06	
	Grade 7 sadness	0.55^***^	0.02	0.54	0.51, 0.60	
Model 3 Adjusted *R*^2^ = 0.37	Gender	−0.25^***^	0.04	−0.13	−0.33, −0.17	0.07
	Age	−0.01	0.19	0.00	−0.38, 0.36	0.00
	Born in Canada	−0.04	0.05	−0.02	−0.14, 0.06	0.00
	Grade 7 sadness	0.51^***^	0.02	0.50	0.47, 0.55	0.75
	Change in home adult relationships	−0.17^***^	0.02	−0.16	−0.22, −0.12	0.12
	Change in school adult relationships	0.00	0.03	0.00	−0.07, 0.06	0.00
	Change in friendships	−0.02	0.02	−0.02	−0.06, 0.02	0.01
	Feeling safe from getting COIVID-19 at school	−0.13^***^	0.03	−0.10	−0.18, −0.08	0.05

*p < 0.05

**p < 0.01

*** *p < 0.001*.

*B, unstandardized regression coefficient; SE, standard error of B; CI, confidence interval for B; β, beta weight; standardized regression coefficient. Gender (girl = 1, boy = 2) and Born in Canada (no = 0, yes = 1)*.

## Discussion

In this study, we sought to examine the experiences of early adolescents during the COVID-19 pandemic, and the pandemic-related factors that were associated with their well-being. Regarding our first objective, 46% of students reported feeling slightly or somewhat worried about their mental/emotional health being influenced by the pandemic in the past 2 weeks. A further 17% reported feeling very or extremely worried. In contrast, 37% of students reported feeling not at all worried about their mental health being influenced by the pandemic in the past 2 weeks. These results align with recent research in Canada that has found wide variation in 10–12 year-olds' self-reported mental health during the pandemic, with the largest proportion of respondents reporting no changes in mental health compared to before the pandemic (44–58%), and another large group (35–46%) reporting that their mental health had worsened (1). Similarly, a multi-wave study of mental health among youth ages 14–28 in Canada identified three latent profile groups that reflected their level of COVID-19-related worries (including the CRISIS measure of worries about their mental/emotional health being influenced by the pandemic). The largest group (52%) reported a moderate level of worries across the first 8 months of the pandemic; the second largest group (35%) reported a low level of worries; the smallest but still sizeable group (13%) reported a high level of worries (54).

In addition to measuring students' self-reported worries about mental health being influenced by the pandemic, we also examined changes in students' well-being and mental health from the year prior to the pandemic to nearly 1 year into the pandemic. On average, students reported significantly lower levels of optimism and life satisfaction and higher levels of sadness during the pandemic compared to the previous academic year, although the difference was small in terms of magnitude of effect sizes. This is consistent with other repeated-measures studies with adolescents 11 to 17 years-old in which participants reported increased depression, anxiety, and decreased life satisfaction during the pandemic compared to their responses before the pandemic (19, 20). It is important to note that declines in well-being commonly occur across this age range (10) and therefore the declines in well-being in our study cannot be attributed solely to the pandemic. However, to put these findings in context, research with older adolescents (i.e., 14 to 28 year-olds) has also found declines in mental health retrospectively compared to before the pandemic, both in clinical and community samples (55). Similarly, matched comparisons between pre-pandemic and pandemic study cohorts found that 11 to 17 year-olds during the pandemic reported significantly higher levels of mental health problems and lower health-related quality of life compared to their same-age pre-pandemic controls (5). Findings from these studies support that declines in students' well-being outcomes in the current sample may have been over and above the declines commonly observed in students transitioning to grade 8.

Our second objective was to examine factors that were associated with students' mental health and resilience. A major finding of this study was that after accounting for student demographics and prior well-being levels, improvements in relationships with parents and other adults at home during the pandemic consistently emerged as one of the most important predictors of students' optimism, life satisfaction, and lower sadness. Early in the pandemic, concerns were raised about the impact of social distancing and pandemic-related stressors on adolescents' mental health and loneliness (27, 56). However, this research also suggested that spending more time together as a result of the pandemic might create opportunities for building stronger relationships within families (27, 56). Subsequent research on this topic has been mixed. For example in one study, adolescents reported no changes in family positive affect or parental warmth compared to before the pandemic (57). In another study, adolescents reported increased family conflicts and deteriorated family climate (5). As noted in these studies, family circumstances and experiences during the pandemic have varied widely (57). In the current study, 85% of students reported that their relationships with parents or adults at home had stayed the same since the pandemic or improved. Although this result is encouraging, it is important to highlight and better understand the contexts of the 15% of adolescents reporting deteriorated relationships with parents and other adults at home. Future research should closely investigate disparities in mental health and relationship impacts of the pandemic, for example through person-centered quantitative analyses and qualitative methods.

Furthermore, related to relationships with adults at home, 29% of our sample reported that spending time with family in-person and virtually had helped them deal/cope with worries and stress related to the COVID-19 pandemic. This aligns with other COVID-19 pandemic research that has identified maintaining a predictable and supportive structure at home to be an important factor in maintaining children's and adolescents' mental health in times of stress (35, 57–59). Specifically, eating meals together, creating family routines, maintaining good communication, engaging in shared activities, and creating a sense of belonging within families have been identified as resilience-building supports for children and adolescents during the pandemic (57–59).

Improvements in other relationships during the pandemic were also associated with higher well-being, although they were relatively not as important according to the Pratt Index. Specifically, relationships with teachers and other adults at school were statistically significantly associated with higher optimism and life satisfaction during the pandemic. Improved relationships with peers were also statistically significantly associated with higher optimism.

It is noteworthy that some students reported that their relationships with adults at home, at school, and with friends improved during a time when well-being generally declines. The associations between relationship improvements and higher well-being observed in this study were consistent with a stress buffering effect of positive relationships (15). These results also align with the PYD framework positing the importance of positive interactions between individuals and supportive environments (16). Feeling safe from getting COVID-19 at school was also consistently associated with higher well-being, although this variable was relatively not as important according to the Pratt Index. We also observed an effect for gender, with boys reporting higher well-being during the pandemic than girls. This finding is consistent with other research finding that girls have reported greater stress and mental health impacts of the pandemic than boys (31). Overall, the results of this study highlight the resilience-promoting role of social relationships for mental health, particularly in times of adversity or crisis.

### Strengths and Limitations

This study had several strengths including the population-level sample within a large urban school district in BC. The timing of the MDI survey prior to the pandemic, and during the pandemic, furthermore provided a rare opportunity to examine longitudinal changes in early adolescents' mental health and well-being, from their own perspectives. Child self-reports are recommended for their accuracy in providing more contextualized information beyond parent measures or teacher-reported assessments that tend to rate children's behaviors more generally (5, 60–62). In this study, new items were developed and adapted specifically to capture early adolescents' lived experiences during the COVID-19 pandemic that were pilot-tested with students and stakeholders from schools and the BC Ministry of Education.

This study also has several limitations that warrant consideration. While our data captured a population sample within a large urban school district, results may not be generalizable to children living in other contexts (i.e., rural or suburban areas, regions outside of BC, Canada). Asking participants to self-assess changes in their mental health status, relationships, and time use compared to before the pandemic using single-item measures may be considered a limitation. However, it is important to note that single-item measures of self-perceived health status have shown evidence for validity as well as sensitivity to change over time in previous research studies (63). Furthermore, where possible we took efforts to directly use or adapt items from previous surveys developed by recognized authorities including the National Institutes of Mental Health (CRISIS) and the BC Centre for Disease Control (41, 49). It would be strategic for future research to examine changes in students' mental health using validated screening measures that can be compared against previous years and other pandemic research.

Use of repeated-measures across the two data collection points was a strength of this study, however without a comparable baseline measure of changes in well-being from Grade 7 to Grade 8 we cannot be certain what proportion of the declines are due to the pandemic vs. child age. That said, comparisons with other pandemic research corroborate that adolescents have reported lower well-being this year than previous years (5, 55). We also took efforts to address this limitation by controlling for child age and Grade 7 well-being in our analyses that examined associations between Grade 8 well-being outcomes and pandemic-related changes in social interactions, activities, and stressors. Another limitation is that we could not control for all potential confounders, for example parent mental health and family socioeconomic status, which may have influenced these associations. Loss to follow up between the two time points furthermore may have introduced sampling bias. We found no demographic differences between children at Grade 7 and those in the linked sample, however our analysis did show that children in the linked sample generally reported higher connectedness to adults and peer belonging than children lost to follow up. Underrepresentation in the linked sample of children with lower adult and peer connectedness may have attenuated the observed associations in this study. Furthermore, missing data on survey items within the linked sample may have introduced information bias in either direction.

## Implications and Conclusions

One of the unique aspects of this study was understanding students' experiences during the COVID-19 pandemic, in the school context, from their own perspectives. Several of the findings have potential interest to educators and administrators tasked with improving school systems during and beyond the pandemic. For example, the majority of students reported that it was a little easy or very easy to follow school safety protocols, with practicing physical distancing and avoiding big groups noted as the most challenging protocols for students. In the context of the school safety protocols, 61% of students felt safe from getting COVID-19 at school most of the time or always. What students have missed at school most during the pandemic are field trips, class parties, and school sports clubs. Students also reported on what has helped them cope with worries and stress related to the pandemic. Most frequently, students reported connecting virtually with friends, exploring interests, spending time outdoors, exercising, connecting with family in-person and virtually, and connecting with friends in-person. Schools may therefore want to prioritize enabling students to socialize safely, and promote time for explored interests and outdoor activities.

With regard to predicting changes in well-being from prior to during the pandemic our results indicate that improvements in relationships with parents and other adults at home during the pandemic consistently emerged as one of the most important predictors of optimism, life satisfaction, and lower sadness, underlining the importance of supportive social relationships.

## Data Availability Statement

The datasets presented in this article are not readily available because the dataset is stored on a Secure Research Environment with Population Data BC (please see https://www.popdata.bc.ca). By regulations of Population Data BC, only individuals added to our research ethics and undergoing training with Population Data BC are allowed to access the data to ensure confidentiality. Requests to access the dataset should be directed to anne.gadermann@ubc.ca.

## Ethics Statement

The studies involving human participants were reviewed and approved by Behavioral Research Ethics Board, University of British Columbia. Written informed consent from the participants' legal guardian/next of kin was not required to participate in this study in accordance with the national legislation and the institutional requirements.

## Author Contributions

AG co-led the conceptualization of the study, directed the project administration, formal analysis, and writing. KT, MGP, MG, MW, and KS-R contributed to the conceptualization of the study, interpretation of analyses, and writing of the manuscript. RG contributed to the conceptualization of the study, conducted the statistical analyses, and contributed to the writing of the manuscript. EO co-led the conceptualization of the study and contributed to the interpretation of analyses and writing of the manuscript. All authors contributed to the article and approved the submitted version.

## Conflict of Interest

The authors declare that the research was conducted in the absence of any commercial or financial relationships that could be construed as a potential conflict of interest.

## Publisher's Note

All claims expressed in this article are solely those of the authors and do not necessarily represent those of their affiliated organizations, or those of the publisher, the editors and the reviewers. Any product that may be evaluated in this article, or claim that may be made by its manufacturer, is not guaranteed or endorsed by the publisher.

## Footnotes

^1^The last survey completed at Time 1 was March 6, just prior to the WHO declaring the COVID-19 pandemic [March 11, 2020 (38)] and the British Columbia Ministry of Education closing schools for two months as a public health safety measure before a partial re-opening in June (29).

^2^Schools were closed to in-person learning in March of 2020 for most students in British Columbia (BC), with voluntary, part-time return to in-person learning open to all students in June of 2020 (29). All students were welcomed back to in-person learning in September 2020, although some families opted to keep children at home, evidenced by a 32% increase in enrolment in Distributed Learning from 2019–2020 to 2020–2021. Schools remained open throughout the 2020–2021 academic year under enhanced health and safety measures (41).

^3^Intraclass correlation coefficients (ICC) were calculated for the three outcome measures to test the proportion of variance that could be explained by clustering in the data by classroom. ICCs for each outcome were below a threshold of 0.10 [Grade 8 optimism (ICC = 0.02), Grade 8 satisfaction with life (ICC = 0.03), Grade 8 sadness (ICC = 0.02)], indicating that single level analyses were appropriate (53).

## References

[1] Cost KT Crosbie J Anagnostou E Birken CS Charach A Monga S. Mostly worse, occasionally better: impact of COVID-19 pandemic on the mental health of Canadian children and adolescents. Eur Child Adolesc Psychiatry. (2021) 1–14. 10.1007/s00787-021-01744-3 33219859

[2] Gassman-Pines A Oltmans Ananat E Fitz-Henley J. COVID-19 and parent-child psychological well-being. Pediatrics. (2020) 146:e2020007294. 10.1542/peds.2020-007294 32764151 PMC7546085

[3] Jones EAK Mitra AK Bhuiyan AR. Impact of covid-19 on mental health in adolescents: a systematic review. Int J Environ Res Public Health. (2021) 18:1–9. 10.3390/ijerph18052470 33802278 PMC7967607

[4] Nearchou F Flinn C Niland R Subramaniam S Hennessy E. Exploring the impact of COVID-19 on mental health outcomes in children and adolescents: a systematic review. Int J Environ Res Public Health. (2020) 17:8479. 10.3390/ijerph17228479 33207689 PMC7698263

[5] Ravens-Sieberer U Kaman A Otto C Erhart M Devine J Schlack R. Impact of the COVID-19 pandemic on quality of life and mental health in children and adolescents. SSRN Electr J. (2020) 94:0–4. 10.2139/ssrn.3721508 34951677 PMC8702614

[6] Wade M Prime H Browne DT. Why we need longitudinal mental health research with children and youth during (and after) the COVID-19 pandemic. Psychiatry Res. (2020) 290:113143. 10.1016/j.psychres.2020.113143 32502829 PMC7253952

[7] Cianfarani S Pampanini V. The impact of stress on health in childhood and adolescence in the era of the COVID-19 pandemic. Horm Res Paediatr. (2021) 345–9. 10.1159/000517460 34261075 PMC8339036

[8] Kessler RC Berglund P Demler O Jin R Merikangas KR Walters EE. Lifetime prevalence and age-of-onset distributions of DSM-IV disorders in the national comorbidity survey replication. Arch Gen Psychiatry. (2005) 62:593–602. 10.1001/archpsyc.62.6.593 15939837

[9] Merikangas KR Nakamura EF Kessler RC. Epidemiology of mental disorders in children and adolescents. Dial Clin Neurosci. (2009) 11:7–20. 10.31887/DCNS.2009.11.1/krmerikangas 19432384 PMC2807642

[10] Schonert-Reichl KA. Middle Childhood Inside and Out : The Psychological and Social Worlds of Canadian Children Ages 9-12, Full Report. The University of British Columbia. (2011).

[11] Eccles J. Schools, academic motivation and stage-environment fit. In: Lerner R Steinberg L editors. Handbook of Adolescent Development. Wiley (2004). pp. 125–53.

[12] Eccles JS Roeser RW. Schools as developmental contexts during adolescence. In: Weiner I editor. Handbook of Psychology. Developmental Psychology. 6th ed. John Wiley and Sons Inc. (2013) pp. 321–37.

[13] Erikson EH. Identity: Youth and Crisis. Norton & Co. (1968).

[14] Klimstra TA Hale WW Raaijmakers QAW Branje SJT Meeus WHJ. Identity formation in adolescence: change or stability? J Youth Adolesc. (2010) 39:150–62. 10.1007/s10964-009-9401-4 20084561 PMC2807933

[15] Hostinar CE Gunnar MR. Future directions in the study of social relationships as regulators of the HPA axis across development. J Clin Child Adoles Psychol. (2013) 42:564–75. 10.1080/15374416.2013.804387 23746193 PMC4161011

[16] Theokas C Almerigi JB Lerner RM Dowling EM Benson PL Scales PC. Conceptualizing and modeling individual and ecological asset components of thriving in early adolescence. J Early Adolesc. (2005) 25:113–43. 10.1177/0272431604272460

[17] Sharma M Idele P Manzini A Aladro CP Ipince A Olsson G. Life in Lockdown: Child and Adolescent Mental Health and Well-Being in the Time of COVID-19. UNICEF (2021).

[18] Vaillancourt T Szatmari P Georgiades K Krygsman A. The impact of COVID-19 on the mental health of Canadian children and youth. Facets. (2021) 6:1628–48. 10.1139/facets-2021-0078 34591373 PMC8646541

[19] Shoshani A Kor A. The mental health effects of the COVID-19 pandemic on children and adolescents : risk and protective factors. Psychol Trauma. (2021). 10.1037/tra0001188 34928689

[20] Magson NR Freeman JYA Rapee RM Richardson CE Oar EL Fardouly J. Risk and protective factors for prospective changes in adolescent mental health during the COVID-19 pandemic. J Youth Adolesc. (2021) 50:44–57. 10.1007/s10964-020-01332-9 33108542 PMC7590912

[21] Korzinski D,. Kids COVID-19: Canadian Children Are Done With School From Home, Fear Falling Behind, Miss Their Friends. Angus Reid Institute (2020). Available online at: http://angus-reid.org/covid19-kids-opening-schools/

[22] Luthar SS Pao LS Kumar NL. COVID-19 and resilience in schools: implications for practice and policy. Social Policy Report. (2021) 34:1–65. 10.1002/sop2.16

[23] Gadermann AM Thomson KC Richardson CG Gagné M Mcauliffe C Hirani S. Examining the impacts of the COVID-19 pandemic on family mental health in Canada : findings from a national cross- sectional study. BMJ Open. (2021) 11:e042871. 10.1136/bmjopen-2020-042871 33436472 PMC7804831

[24] Westrupp EM Bennett C Berkowitz T Youssef GJ Toumbourou JW Tucker R. Child, parent, and family mental health and functioning in Australia during COVID-19: comparison to pre-pandemic data. Eur Child Adolesc Psychiatry. (2021) 1–14. 10.1007/s00787-021-01861-z 33219859

[25] Lee SJ Ward KP Chang OD Downing KM. Parenting activities and the transition to home-based education during the COVID-19 pandemic. Children Youth Serv Rev. (2020) 122:105585. 10.1016/j.childyouth.2020.105585 33071407 PMC7553006

[26] Lee SJ Ward KP Lee JY Rodriguez CM. Parental social isolation and child maltreatment risk during the COVID-19 pandemic. J Fam Violence. (2021) 1–12. 10.1007/s10896-020-00244-3 33462526 PMC7807402

[27] Cluver L Lachman JM Sherr L Wessels I Krug E Rakotomalala S. Parenting in a time of COVID-19. Lancet. (2020) 395:e64 10.1016/S0140-6736(20)30736-4 32220657 PMC7146667

[28] Fegert JM Vitiello B Plener PL Clemens V. Challenges and burden of the Coronavirus 2019 (COVID-19) pandemic for child and adolescent mental health: a narrative review to highlight clinical and research needs in the acute phase and the long return to normality. Child Adolesc Psychiatry Ment Health. (2020) 14:1–11. 10.1186/s13034-020-00329-3 31956339 PMC6958641

[29] Dove N Wong J Gustafson R Corneil T. Impact of School Closures on Learning, Child Family Well-Being During the COVID-19 Pandemic (Issue September). (2020). Available online at: http://www.bccdc.ca/Health-Info-Site/Documents/Public_health_COVID-1 (accessed December 15, 2021).

[30] BC Centre for Disease Control - BC Ministry of Health *COVID-19 Public Health Guidance for K-12 Schools: Vol. February*. (2021). Available online at: http://www.bccdc.ca/Health-Info-Site/Documents/COVID_public_guidance/Guidance-k-12-schools.pdf

[31] Schwartz KD Exner-Cortens D McMorris CA Makarenko E Arnold P Van Bavel M. COVID-19 and student well-being: stress and mental health during return-to-school. Can J Sch Psychol. (2021) 36:166–85. 10.1177/08295735211001653 34040284 PMC8114331

[32] Human Early Learning Partnership. How are the Kids? Children's Perspectives on their Health, Well-Being and Assets both Before and During a Global Pandemic through the Middle Years Development Instrument (MDI) in British Columbia, Canada (2021).

[33] Masten AS Tellegen A. Resilience in developmental psychopathology: contributions of the Project Competence Longitudinal Study. Dev Psychopathol. (2012) 24:345–61. 10.1017/S095457941200003X 22559118

[34] Ellis WE Dumas TM Forbes LM. Physically isolated but socially connected: psychological adjustment and stress among adolescents during the initial COVID-19 crisis. Can J Behav Sci. (2020) 52:177–87. 10.1037/cbs0000215

[35] McArthur BA Racine N McDonald S Tough S Madigan S. Child and family factors associated with child mental health and well-being during COVID-19. Eur Child Adolesc Psychiatry. (2021) 1–11. 10.1007/s00787-021-01849-9 33219859

[36] Kerekes N Bador K Sfendla A Belaatar M El Mzadi A Jovic V. Changes in adolescents' psychosocial functioning and well-being as a consequence of long-term covid-19 restrictions. Int J Environ Res Public Health. (2021) 18:8755. 10.3390/ijerph18168755 34444502 PMC8392883

[37] Statistics Canada. Census Profile. *2016 Census* (2016).

[38] World Health Organization. Timeline: WHO's COVID-19 Response. (2021). Available online at: https://www.who.int/emergencies/diseases/novel-coronavirus-2019/interactive-timeline#!

[39] Guhn M Schonert-Reichl KA Gadermann AM Marriott D Pedrini L Hymel S. Well-being in middle childhood: an assets-based population-level research-to-action project. Child Indic Res. (2012) 5:393–418. 10.1007/s12187-012-9136-8

[40] Schonert-Reichl KA Guhn M Gadermann AM Hymel S Sweiss L Hertzman C. Development and validation of the Middle Years Development Instrument (MDI): assessing children's well-being and assets across multiple contexts. Soc Indic Res. (2013) 114:345–69. 10.1007/s11205-012-0149-y 24109151 PMC3790250

[41] British Columbia Centre for Disease Control. COVID-19 SPEAK Survey. (2021). Available online at: http://www.bccdc.ca/health-info/diseases-conditions/covid-19/covid-19-survey

[42] Castelli L Marcionetti J Crescentini A Sciaroni L. Monitoring preadolescents' well-being: Italian validation of the middle years development instrument. Child Indic Res. (2018) 11:609–28. 10.1007/s12187-017-9459-6

[43] Gregory T Engelhardt D Lewkowicz A Luddy S Guhn M Gadermann A. Validity of the middle years development instrument for population monitoring of student wellbeing in Australian school children. Child Indicators Res. (2018) 6:695–708. 10.1007/s12187-018-9562-3

[44] Noam GG Goldstein LS. The Resilience Inventory (1998).

[45] Gadermann AM Schonert-Reichl KA Zumbo BD. Investigating validity evidence of the satisfaction with life scale adapted for children. Soc Indic Res. (2010) 96:229–47. 10.1007/s11205-009-9474-1

[46] Gadermann AM Guhn M Zumbo BD. Investigating the substantive aspect of construct validity for the satisfaction with life scale adapted for children : a focus on cognitive processes. Soc Indic Res. (2011) 100:37–60. 10.1007/s11205-010-9603-x

[47] Diener E Emmons RA Larsen RJ Griffin S. The satisfaction with life scale. J Pers Assess. (1985) 49:71–75. 10.1207/s15327752jpa4901_13 16367493

[48] Kusche CA Greenberg MT Beilke R. Seattle personality questionnaire for young school-aged children. Department of Psychology, University of Washington, Seattle, United States (1988).

[49] Nikolaidis A Paksarian D Alexander L Derosa J Dunn J Nielson DM. The Coronavirus Health and Impact Survey (CRISIS) reveals reproducible correlates of pandemic-related mood states across the Atlantic. Sci Rep. (2021) 11:1–13. 10.1038/s41598-021-87270-3 33414495 PMC7791137

[50] Mental Health Foundation (UK). The COVID-19 Pandemic, Financial Inequality and Mental Health: A Briefing From the “Coronavirus: Mental Health in the Pandemic” Study. (2020).

[51] Thomas D Roland Hughes E Zumbo BD. On variable importance in linear regression. Soc Indic Res. (1998) 45:253–75. 10.1023/A:1006954016433

[52] Thomas DR. Interpreting discriminant functions: a data analytic approach. Multivariate Behav Res. (1992) 27:355–62. 10.1207/s15327906mbr2703_3 26789787

[53] Irimata KM Wilson JR. Identifying intraclass correlations necessitating hierarchical modeling. J Appl Stat. (2018) 45:626–41. 10.1080/02664763.2017.1288203

[54] Hawke LD Szatmari P Cleverley K Courtney D Cheung A Voineskos AN. Youth in a pandemic: a longitudinal examination of youth mental health and substance use concerns during COVID-19. BMJ Open. (2021) 11:e049209. 10.1136/bmjopen-2021-049209 34716160 PMC8561825

[55] Hawke LD Barbic SP Voineskos A Szatmari P Cleverley K Hayes E. Impacts of COVID-19 on youth mental health, substance use, and well-being: a rapid survey of clinical and community samples. Can J Psychiatry. (2020) 65:701–9. 10.1177/0706743720940562 32662303 PMC7502874

[56] Loades ME Chatburn E Higson-Sweeney N Reynolds S Shafran R Brigden A. Rapid systematic review: the impact of social isolation and loneliness on the mental health of children and adolescents in the context of COVID-19. J Am Acad Child Adolesc Psychiatry. (2020) 59:1218–39.e3. 10.1016/j.jaac.2020.05.009 32504808 PMC7267797

[57] Janssen LHC Kullberg ML Verkuil B van Zwieten N Wever MCM van Houtum. Does the COVID-19 pandemic impact parents' and adolescents' well-being? An EMA-study on daily affect and parenting. PLoS ONE. (2020) 15:1–21. 10.1371/journal.pone.0240962 33064778 PMC7567366

[58] Gayatri M Irawaty DK. Family resilience during COVID-19 pandemic: a literature review. Family J. (2021) 1. 10.1177/10664807211023875 35399750 PMC8980846

[59] Glynn LM Davis EP Luby JL Baram TZ Sandman CA. A predictable home environment may protect child mental health during the COVID-19 pandemic. Neurobiol Stress. (2021) 14:100291. 10.1016/j.ynstr.2020.100291 33532520 PMC7823041

[60] De Los Reyes A Kazdin AE. Informant discrepancies in the assessment of childhood psychopathology: a critical review, theoretical framework, and recommendations for further study. Psychol Bull. (2005) 131:483–509. 10.1037/0033-2909.131.4.483 16060799

[61] Goodman R. Psychometric properties of the strengths and difficulties questionnaire. J Am Acad Child Adolesc Psychiatry. (2001) 40:1337–45. 10.1097/00004583-200111000-00015 11699809

[62] Ravens-sieberer U Devine J Bevans K Riley AW Moon J Salsman JM. Subjective well-being measures for children were developed within the PROMIS project : presentation of first results. J Clin Epidemiol. (2014) 67:207–18. 10.1016/j.jclinepi.2013.08.018 24295987 PMC4120943

[63] Macias C Gold PB Öngür D Cohen BM Panch T. Are single-item global ratings useful for assessing health status? J Clin Psychol Med Settings. (2015) 22:251–64. 10.1007/s10880-015-9436-5 26492891

