# Supplementary material for: Early Adolescents' Experiences During the COVID-19 Pandemic and Changes in Their Well-Being
Source: Front Public Health. 2022 May 3;10:823303. doi: 10.3389/fpubh.2022.823303 (PMC9110968; doi:10.3389/fpubh.2022.823303)
Supplement: Supplementary file 1 [file Data_Sheet_1.PDF]

Appendix A: COVID-19 Pandemic Module Items from the Middle Years Development Instrument

The last 18 questions ask about your experiences during the Coronavirus/COVID-19 pandemic. Some of these questions ask how your life is different now compared to before the Coronavirus/COVID-19 pandemic.

As a reminder, you don't have to answer any questions you don't want to and you can skip any questions. You can withdraw from the survey at any time by clicking on the Withdraw from this survey button at the bottom of every page. It is important for you to know that ALL OF YOUR ANSWERS that you put in this survey will be confidential (private) and will not be shared with your teacher, principal, parents, or your friends.

Compared to before the COVID-19 pandemic

How did the following change from before the COVID-19 pandemic to now?

	Much Less	Less	About the same	More	Much more
102. I spend time with my friends online.	◯	◯	◯	◯	◯
103. I spend time with my friends in person/face-to-face.	◯	◯	◯	◯	◯
104. I spend time with family members who live in my home.	◯	◯	◯	◯	◯
105. I spend time outdoors (for example, playing sports, hiking, biking, going for walks).	◯	◯	◯	◯	◯

Previous   Next

SAMPLE ONLY NOT FOR USE

From before the COVID-19 pandemic to now...

	A lot worse	A little worse	About the same	A little better	A lot better
106. ... have your relationships with your <u>parents or other adults at home</u> gotten worse or better?	○	○	○	○	○
107. ... have your relationships with <u>teachers or other adults at your school</u> gotten worse or better?	○	○	○	○	○
108. ... have your relationships with your <u>friends</u> gotten worse or better?	○	○	○	○	○

Previous

Next

SAMPLE ONLY
NOT FOR USE

During the COVID-19 pandemic

	Never	Rarely	Occasionally	Often	Most of the time
109. How much are you reading or talking about the COVID-19 pandemic?	○	○	○	○	○

	Not at all	Slightly	Somewhat	Very	Extremely
110. During the PAST TWO WEEKS, how worried have you been about your Mental/Emotional health being influenced by the COVID-19 pandemic?	○	○	○	○	○

Previous

Next

SAMPLE ONLY
NOT FOR USE

111. What has helped you deal/cope with worries and stress related to the COVID-19 pandemic? ***(Please select all the things that have helped you deal/cope with worries and stress.)***

- ☐ I have not felt worried or stressed much about the COVID-19 pandemic
- ☐ Connecting in-person with family
- ☐ Connecting virtually with family (phone, video games/chat, text)
- ☐ Connecting in-person with friends
- ☐ Connecting virtually with friends (phone, video games/chat, text)
- ☐ Getting support from my teacher(s) or other adults at school (principal, counsellor)
- ☐ Getting support from a counsellor or therapist outside of school
- ☐ Accessing mental health websites or apps (for example, KidsHelpPhone)
- ☐ Exercising
- ☐ Spending time outdoors
- ☐ Spending time with pet(s)
- ☐ Exploring my interests (music, reading, arts & crafts)
- ☐ Volunteering to help
- ☑ Other
- ☐ Nothing has helped me deal/cope with worries and stress related to the COVID-19 pandemic

Other (describe): ____________________

Previous | Next

SAMPLE ONLY
NOT FOR USE

How easy or hard is it for you to do the following behaviours at your school?

	Very hard	A little hard	Not hard or easy	A little easy	Very easy	Does not apply (we don't do this at my school)
112. Wear a mask	○	○	○	○	○	○
113. Practice physical distancing	○	○	○	○	○	○
114. Wash hands (or use hand sanitizer) more often	○	○	○	○	○	○
115. Stay home when sick	○	○	○	○	○	○
116. Avoid big groups	○	○	○	○	○	○

SAMPLE ONLY
NOT FOR USE

Previous

Next

Many changes have been made to protect children from getting COVID-19 at school.

117. How often do the COVID-19 safety measures at school make you feel safe from getting COVID-19?	Never safe	Safe some of the time	Safe most of the time	Always safe
	○	○	○	○

Previous

Next

SAMPLE ONLY
NOT FOR USE

118. What do you miss the most at school during the COVID-19 pandemic? ***(Please select all the things you miss the most.)***

- [ ] Field trips
- [ ] Assemblies
- [ ] School sports clubs (for example, cross-country, volleyball, basketball, soccer)
- [ ] Other school clubs (for example, environmental club, arts club)
- [ ] Choir, strings, band
- [ ] Little buddies - reading program with younger grades
- [ ] Lunch monitor/Park monitor
- [ ] Going to library during free time
- [ ] Class parties
- [ ] Typical instruction (the ways you used to learn before the pandemic)
- [x] Other things I miss most at school

Other (describe): ______________________

Previous   Next

SAMPLE ONLY
NOT FOR USE

119. What are some <u>positive changes</u> that have occurred in your life during the COVID-19 pandemic? ***(Please select all the positive changes that have occurred.)***

- [ ] There were <u>no</u> positive changes
- [ ] Exploring other interests (for example, cooking, learning a new instrument, learning a new language, eating new foods)
- [ ] Spending more time with friends
- [ ] Spending more time with family members (for example, parents/guardians, siblings)
- [ ] Getting more sleep
- [ ] Having more time to yourself
- [ ] Helping others more (for example, walking your neighbour's dog, helping prepare food)
- [x] Other

Other (describe): ____________

Previous   Next

SAMPLE ONLY
NOT FOR USE
